# Armor-like chronic actinic dermatitis

**DOI:** 10.11604/pamj.2025.50.114.39129

**Published:** 2025-04-28

**Authors:** Xinrong Chen, Jie Chen

**Affiliations:** 1 Zhuji People's Hospital of Zhejiang Province, Shaoxing 311800, China

**Keywords:** Chronic actinic dermatitis, armor-like, hypersensitivity

## Image in medicine

The pathogenesis of chronic actinic dermatitis is not well understood, which is speculated to be a delayed hypersensitivity caused by light induction. It is found that immunological basis is involved, and the infiltrating cells in the dermis are mainly CD8+ cytotoxic inhibitory T cells. They tend to occur in areas exposed to sunlight. A 68-year-old male presented with erythema infiltrating plaques on the frontal face for more than 5 years with pruritus. The skin shaped like armor, was diagnosed with armor-like chronic actinic dermatitis. The differential diagnoses may include seborrheic dermatitis, dermatomyositis, contact dermatitis. Topical use of a strong hormone cream may relieve symptoms, but the lesions are not completely resolved.

**Figure 1:**
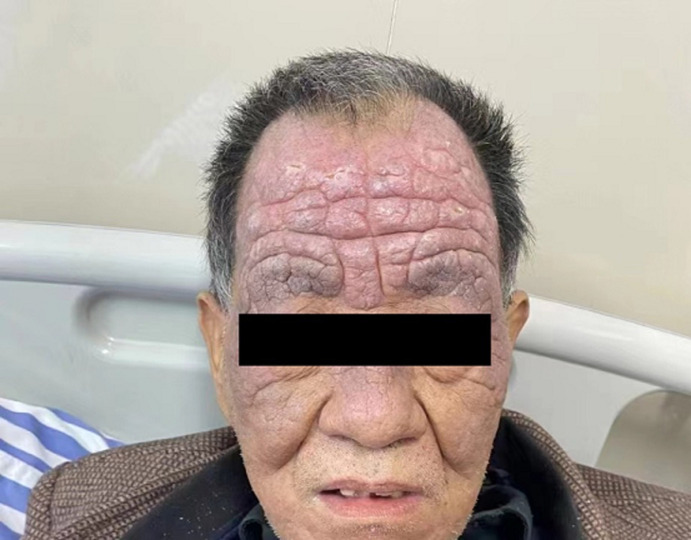
armor-like chronic actinic dermatitis of the frontal face

